# Effect of transcranial direct current stimulation on postpartum depression: A study protocol for a randomized controlled trial

**DOI:** 10.3389/fpsyg.2023.990162

**Published:** 2023-02-15

**Authors:** Weiming Sun, Xizhen Kang, Xiangli Dong, Zijian Zeng, Qing Zou, Meixiang Su, Ke Zhang, Guanxiu Liu, Guohua Yu

**Affiliations:** ^1^Department of Rehabilitation Medicine, The First Affiliated Hospital of Nanchang University, Nanchang, Jiangxi, China; ^2^The First Clinical Medical School, Nanchang University, Nanchang, Jiangxi, China; ^3^Department of Psychosomatic Medicine, The Second Affiliated Hospital of Nanchang University, Nanchang, Jiangxi, China; ^4^Department of Psychosomatic Medicine, The First Affiliated Hospital of Nanchang University, Nanchang, Jiangxi, China; ^5^Nanchang Key Laboratory of Medical and Technology Research, Nanchang University, Nanchang, Jiangxi, China

**Keywords:** transcranial direct current stimulation, postpartum depression, randomized controlled trial, study protocol, mental health

## Abstract

Postpartum depression (PPD) is a complex combination of physiological, emotional, and behavioral alterations associated with postpartum chemical, social, and psychological variations. It does harm to the relationship between family members that could potentially last for years. However, standard depression treatments are not ideal for PPD, and the outcomes of these treatments are debatable. Transcranial direct current stimulation (tDCS) is an emerging technology that could provide patients with PPD with a safe and non-pharmacological treatment. tDCS can relieve depression by directly stimulating the prefrontal cortex through the excitatory effect of the anode. It may also ease depression indirectly by promoting the production and release of the neurotransmitter GABA. The mechanism of tDCS makes it an ideal therapeutic approach to treat PPD, although it has not been widely used, and its effect has not been evaluated systematically and effectively. A double-blind, randomized controlled trial will be conducted involving 240 tDCS-naive patients with PPD, who will be randomly divided into two groups. One group will receive routine clinical treatment and care with active tDCS, and the other group will receive routine clinical treatment and care with sham tDCS. Each group of patients will receive a 3-week intervention during which they will receive 20 min of active or sham tDCS 6 days per week. The Montgomery–Åsberg Depression Rating Scale will be administered before the intervention as a baseline and on each weekend throughout the intervention phase. Before and after the intervention, the Perceived Stress Scale and the Positive and Negative Affect Schedule will be evaluated. Side effects and abnormal reactions will be recorded during each treatment. As antidepressants are banned in the study, the results will not be affected by drugs and will therefore be more accurate. Nonetheless, this experiment will be conducted in a single center as a small sample experiment. Therefore, future studies are required to confirm the effectiveness of tDCS in treating PPD.

## 1. Introduction

Postpartum depression (PPD) is a complex combination of physiological, emotional, and behavioral alterations associated with postpartum chemical, social, and psychological variations (Wang et al., 2021). The postpartum period begins after childbirth and lasts up to 12 months after the delivery. PPD can result in significant financial losses annually. Globally, 67% of financial resources are directly allocated to psychiatric health services. A recent review suggested that the global prevalence of PPD was approximately 17% (Wang et al., 2021). This prevalence level means that PPD may significantly affect the wellbeing of parents, the relationship between mothers and their children, cognitive development, social abilities, and behavioral outcomes among children (Nilova et al., 2017). The high incidence rate of maternal mental health disorders presents a risk not only to mothers but also to children and other associated family members. People who are married and suffer from PPD are more likely to separate and divorce, mistreat and neglect their children, experience chronic mental disorders, die by suicide, and commit infanticide (Wisner et al., 2006; Field, 2011; Beestin et al., 2014; Stein et al., 2014; The American College of Obstetricians Gynecologists Committee Opinion no. 630., 2015; Punescu et al., 2017). Numerous studies (Cheng et al., 2016; Closa-Monasterolo et al., 2017; Koutra et al., 2017; Van der Waerden et al., 2017) show that PPD results in decreased cognitive function and increased behavioral problems among infants and children. If left untreated, the manifestations of PPD can last for years. It has been calculated that approximately 30% of women suffer from PPD in the community, with the percentage rising to 50% in clinical settings (Goodman, 2004; Vliegen et al., 2014). Therefore, the appropriate treatment of PPD is crucial to ensuring harmony among families and enhancing the relationship between mother and child.

The treatment of depression typically relies on psychotherapeutic methods, such as cognitive-behavioral therapy (CBT), interpersonal psychotherapy (IPT), and social psychotherapy, which can be combined with antidepressants, such as tricyclic antidepressants or selective 5-hydroxytryptamine reuptake inhibitors. Nevertheless, it remains controversial whether the conventional treatment approaches for PPD are effective based on the effects of the treatment approaches mentioned above (Orsolini and Bellantuono, 2015; Uguz, 2016; Thomson and Sharma, 2017). It takes time for psychotherapy to be effective, and it is generally ineffective in cases of major depression. Furthermore, barriers to participation in psychotherapy include perceived stigmatization, the lack of trained therapists specializing in IPT or CBT, the investment of time necessary, as well as the need to arrange childcare needs and its associated costs (Li et al., 2020). While antidepressants may be effective within a few weeks, the increased risk of adverse consequences in children due to fetal exposure has led mothers to refuse to use them while nursing (Sorenson, 1990). However, antidepressants are clinically appropriate for women with severe mental disorders during pregnancy and postpartum, when there are significant risks to the mother, fetus, or baby (Howard et al., 2014). Individuals with PPD may experience increased levels of anxiety when starting an antidepressant-based treatment approach, with previous studies showing that such patients consistently deny medication or are forced to continue with low doses (Boath et al., 2004; Guille et al., 2013). Therefore, the low acceptability of existing treatment approaches may result in the inadequate treatment of depression in this group. Regarding long-term treatment options, it is imperative to explore effective alternatives to treating PPD without adverse side effects. Currently, such alternatives remain inadequate.

Non-invasive brain stimulation (NIBS) is a non-traumatic, convenient, safe, and effective brain function therapy technique widely used in clinics. As a typical NIBS approach, transcranial direct current stimulation (tDCS) may be an effective brain stimulation medical technique with the potential to be an “optimal” treatment method for PPD. Additionally, tDCS, a local brain stimulation-based treatment approach, is dependent on the presence of abnormalities in the activity of the frontal lobe region of the brain, particularly the left and right dorsolateral prefrontal cortex (PFC) (George et al., 1997; Speer et al., 2000; Fitzgerald et al., 2003; Ardolino et al., 2005). In a meta-analysis of 10 studies, researchers found that, compared to sham tDCS, active tDCS was more favorable in alleviating symptoms of depression after 2 to 3 weeks of treatment (Kalu et al., 2012). Significantly, tDCS has proven to be safe in a wide range of trials. In one study involving the side effects of tDCS, 77 healthy controls and 25 patients with neurological diseases were examined 567 times, with the most prevalent side effects involving slight tingling, moderate fatigue, and mild tickling under the stimulation electrodes. After tDCS, approximately 10% of headaches and <3% of instances of insomnia and nausea were reported (Poreisz et al., 2007). Additionally, tDCS is an available treatment approach. The instrumentation required is cheap and mobile, and tDCS can be administered by trained technicians or by the individual themselves.

In summary, tDCS is a novel, safe, and non-pharmacological treatment technique for individuals suffering from PPD. The purpose of this protocol is to evaluate the efficacy and safety of tDCS as a method of treating PPD with a forward-looking, double-armed, sham-control, randomized controlled trial (RCT) protocol toP. This may be a guide to a larger multi-centered RCT that explicitly values the therapeutic effects of tDCS on the treatment of PPD. This study protocol may also be used to derive the initial impact dimensions for the sample size required to complete the most complete and reliable RCT.

## 2. Method

### 2.1. Study design

The RCT will involve recruiting patients with PPD for up to 1–2 years from professional postpartum psychological health projects in the Departments of Psychosomatic Medicine, Rehabilitation Medicine, Obstetrics, and Gynecology at the First Affiliated Hospital of Nanchang University. To recruit enough participants, leaflets and posters will be posted at the place of recruitment, and physicians will be informed about this study to assist in identifying qualified participants. The research coordinator conducted a baseline visit before the start of the active study to collect information on baseline demographics, to gain access to health services, and to gather baseline Montgomery-Asberg Depression Rating Scale (MADRS) measures.

In addition to the intervention, sham control will also be carried out within the Core Clinical Experiment at the first Affiliated Hospital of Nanchang University by well-trained researchers to guarantee proper supervision throughout the procedures. The active period of the experiment involving altogether 18 intervention or sham control sessions lasting for 20 min for approximately 3 weeks (6 days per week). The researchers who manage the session will record the date of every visit, the participant's blood pressure and heart rate, the starting and finishing times of sham tDCS, and interruptions, if any. All confounding factors (such as a previous history of depression, treatments, family history, etc.) will be analyzed. Outcome questionnaires will be collected from patients with PPD at three time points: before the procedures after enrollment (T1), 1 week after completing the procedures (T2), and 3 weeks after completing the procedures (T3). Follow-up questionnaires will be completed in person or by phone 8 weeks after completing the procedures. Patients will receive email updates concerning enrollment goals and the integrity of follow-up to help them to perceive the contributions that they made while participating in the study. Participants are then contacted with outcome information gathered and organized by professionally trained and experienced researchers. Personal outcome information is blind to the researchers, participants, and physicians who provided clinical treatment and care in the study group assignment.

**Figure 1:**
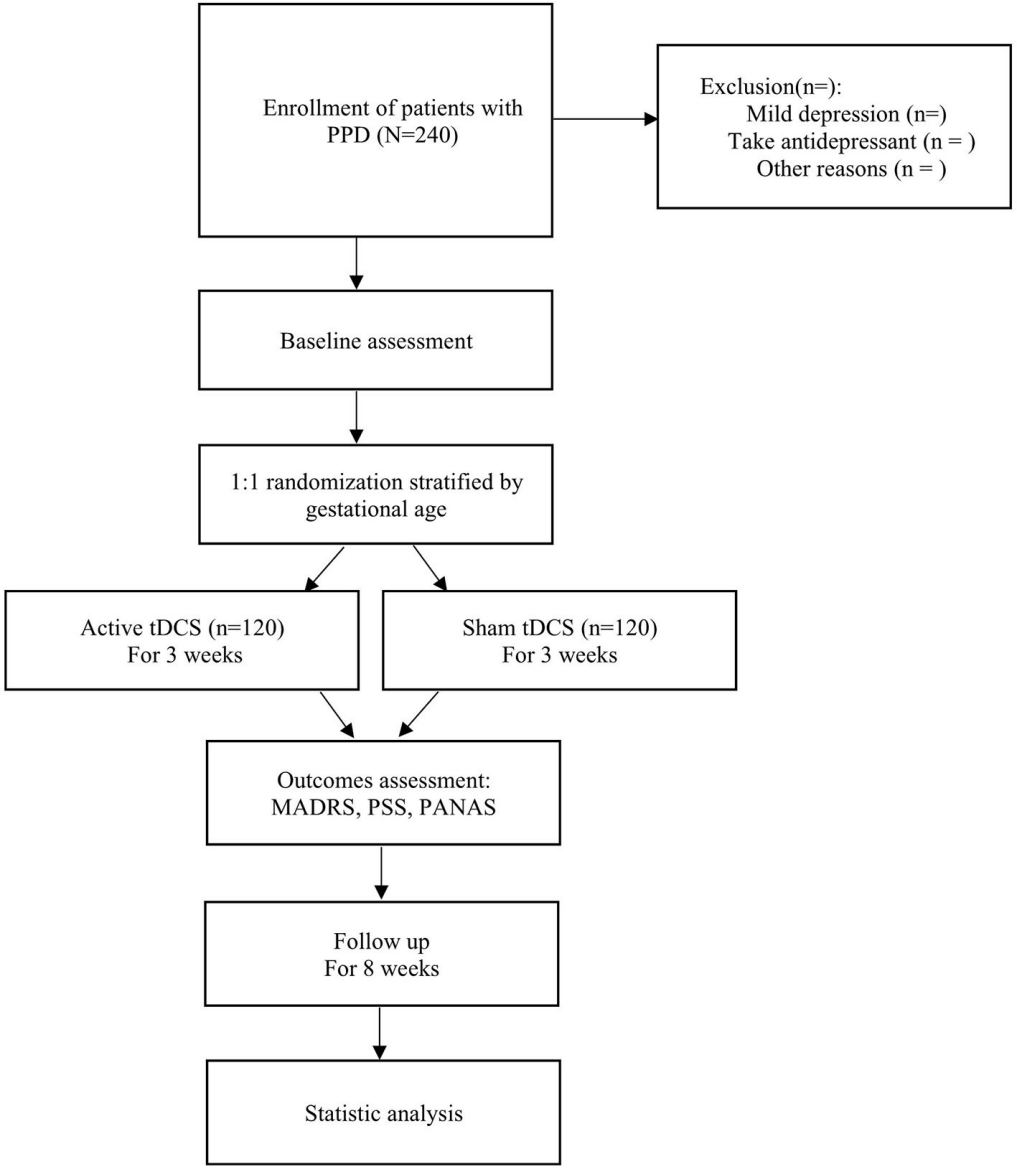
A flow diagram of the trial design. First, eligible patients with PPD are recruited and a baseline assessment is conducted. Then, patients are divided into the intervention and control groups (active tDCS or sham tDCS) randomly and research is implemented for 3 weeks, and outcomes are recorded timely. Finally, patients are followed up for 8 weeks for statistical analysis.

The flow diagram of this study design is shown in Figure 1, and the evaluation of different time points is shown in Table 1.

**Table 1:** Data on the design of the study.

**Point in time**		**Screening stage**	**Treatment period 1 (week 1)**	**Treatment period 2 (week 2)**	**Treatment period 3 (week 3)**	**Follow-up (8 weeks)**
	Informed consent	×				
Enrolment	Inclusion and exclusion criteria	×				
	Basic information	×				
	Past medical history	×				
	Withdrawal, drop out, and termination criteria		×	×	×	×
Intervention	Therapeutic parameter record		×	×	×	×
Assessment	Montgomery-Åsberg Depression Rating Scale (MADRS)	×	×	×	×	×
	Perceived Stress Scale (PSS)	×	×	×	×	×
	Positive and Negative Affect Schedule (PANAS)	×	×	×	×	×
	Overall efficacy evaluation		×	×	×	×
	Complications		×	×	×	×
	Adverse events recorded		×	×	×	×

Corresponding assessments are recorded at five different time points.

### 2.2. Participants and recruitment

Potentially eligible participants will be recruited through the Internet, leaflets, and posters, and preliminary screening will be conducted by trained psychologists, rehabilitation physicians, and obstetricians or gynecologists on visits to the First Affiliated Hospital of Nanchang University. The researchers will then conduct further screening based on eligibility and exclusion criteria to identify eligible participants. Finally, all the participants will provide written informed consent.

### 2.3. Eligibility criteria

To be included in the study, the participants must meet the following criteria:

(1) Be postpartum and at least 18 years old;

(2) Satisfy the current criteria for the diagnosis of severe depression or at least a severe depression paroxysm of moderate severity based on the fifth edition of the Diagnostic and Statistical Manual of Mental Disorders (DSM-5);

(3) Have been provided with and have refused to take antidepressants prescribed by their attending clinicians at the recruitment location;

(4) Be evaluated to qualify for outpatient psychiatric treatment by a chief researcher; and

(5) Have never received tDCS but agree to this treatment approach and comprehend the interpretations of the study and the associated questionnaires.

### 2.4. Exclusion criteria

Participants will be excluded from the study if they

(1) Fail to meet the criteria for the diagnosis of severe depression or the severe depression paroxysm of less than moderate severity at the time of the study;

(2) Have been diagnosed with alcohol or substance use disorder by the DSM-5 within the last 6 months;

(3) Have severe and active medical or neurologic disorders or a history of epilepsy;

(4) Are taking carbamazepine (because it might reduce the effectiveness of tDCS therapy), benzodiazepines, or other anticonvulsants daily at the time of the study;

(5) Have metal objects into the skull, electrically transplanted into the body, or transplanted to the scalp to stimulate the skin at the skin defect; and

(6) Have serious physical diseases or complications cause the patients can't endure the tDCS stimulation.

### 2.5. Sample size

The sample size involved in this study will be based on that of previous studies. The improvements will be evaluated using identical sample sizes based on the following estimated formula:

$\eta =\frac{2\bar{pq}{\left(Z_{\alpha }+Z_{\beta }\right)}^{2}}{{\left(p_{1}-p_{2}\right)}^{2}}$. The first type of error is 5% (α = 0.05) and 90% power (β = 0.10), and the estimated sample size requires 79 participants per group. Given the high drop-out rate (~15–20%) in the study (Lever Taylor et al., 2016), at least 198 participants will be required to achieve the target of 79 participants in each group. We receive approximately 200 patients with PPD per month, combined with approximately 120 patients suffering from moderate to major depressive disorders. According to the recruitment approach of a previous study (Walton et al., 2014), we estimate that 60 patients (50%) may be ineligible owing to antidepressant-based treatment 15 patients may be ineligible for various reasons such as having severe physical diseases or complications, and 20 patients within the remaining 45 patients (50%) may agree to participate in the preliminary study. Therefore, the enlisting of 240 patients suffering from PPD could be achieved within a year.

### 2.6. Randomization and allocation concealment

Following the informed consent process and the assortment of baseline information, individuals suffering from PPD who meet the eligibility criteria pertinent to this study will randomly receive active or sham tDCS. A study aide outside the confines of this study will present an assignment sequence using a randomized table of arranged blocks, and group distributions will be put into air-tight envelopes. The randomization identification numbers (RIDs) in the table will be stratified by the age of the newborn baby at enrollment to balance the gestational age of each group. Therefore, the first layer of RIDs will be distributed to participants who are <3 weeks into the postpartum period at the time of registration. The second layer of RIDs will be distributed to participants who are 3 weeks or more into the postpartum period. The experiment coordinator, while remaining blind to the group assignments, will select an envelope to include in the participants' chart. The experimenter will then open the envelope to ascertain the packet assignment, and program the tDCS device accordingly. The researchers will document the outcome measures objectively. The participants and researchers collecting maternal and infant clinical outcome data will also remain blind to the group distributions.

### 2.7. Blinding

As this will be a double-blind study, a third party not involved in the experiment will administer and supervise the blind strategies as follows:

(1) Participants will not be permitted to open the envelopes in the order in which they will be involved in the study. Assuming a tDCS model of modes A and B, the study implementer will not have any knowledge of what the stimuli represented.

(2) The A model represents an active stimulus, and the B model represents a sham stimulus. A third-party evaluator will evaluate the treatment results without information regarding the subgroup.

(3) The first non-blind method will be conducted until the applied mathematical analysis is completed to overcome subjective tendencies among the analysts during the data analysis process. After statistical analysis, a second non-blinded test will be conducted to determine the intervention group.

(4) Participants will be randomly asked whether they received an active or sham stimulus to ensure that they do not recognize the stimulation pattern.

### 2.8. Ethics issues

This study has been approved by the Ethics Committee of the First Affiliated Hospital of Nanchang University: Clinical Medicine Ethics Review [2015]043.

### 2.9. Dissemination plan

This study aims to investigate the possibility of conducting a multi-centered RCT to assess the effectiveness of tDCS in the treatment of PPD. Therefore, the objectives of the dissemination plan associated with this study are as follows: (1) offering insight for future studies to provide evidence for a multi-centered study examining the efficacy of tDCS in the treatment of PPD and (2) raising awareness and interest among the community and public regarding the potential possibility of this treatment approach in preventing unfavorable maternal and infant outcomes. This approach can facilitate the overcoming of hindrances to treatment satisfaction by ensuring that a clear and impartial message is delivered to potential treatment users, thereby encouraging effective and timely decisions regarding treatment. The group involved in this study is linked to clinical brain stimulation programs, and retraining on the use of tDCS procedures can be offered. Study findings will be presented in peer-reviewed publications to facilitate the rationality of our outcomes. Additionally, individuals suffering from PPD, as well as their families, will be informed of the outcomes in an encouraging and non-stigmatizing manner, ensuring that we facilitate the mass interest in participating in larger treatment trials and the subsequent adoption of this treatment approach.

### 2.10. Informed consent procedures

Participants will be provided with a clear explanation of the purpose, process, risks, and benefits of this study, and all questions asked by participants with the intention of confirming their understanding of the study, the risks and latent benefits of being involved in the study, and their privileges as study subjects will be answered before they sign the consent papers. Patients are required to sign an informed consent document before participating in the study. Informed consent should be obtained prior to any research evaluation or procedures, and before any private information is recorded. During the study period, participants will have an opportunity to agree to continue using the study protocol. We will also request that subjects invite their family and friends to participate in an initial discussion about the study so that all individuals involved in deciding whether to enroll in the study have adequate information. We will guarantee privacy protection of personal information.

### 2.11. Interventions

The intervention group will receive active tDCS, as well as clinical treatment and nursing care. The control group will receive sham tDCS as well as clinical treatment and nursing care. The treatment will be administered six times per week for 3 weeks. Regarding quality management, all the processes involving associated protocols, treatment approaches, and assessments will be standardized and conducted by the therapists participating in this study.

#### 2.11.1. Basic treatment

Patients with PPD will continue to receive clinical treatment and nursing care from their individual clinical programs throughout the trial. Their clinical treatment and nursing suppliers are will be blind to their group assignment. Clinical treatment and nursing care will not include the offer of antidepressants. If participants start taking antidepressants during the study, it will be recorded, and they will not be able to continue participating in the study. The record of antidepressants is shown in Table 2.

**Table 2:** The records of patients with PPD taking antidepressants.

	**Antidepressants type**	**Dosage**	**Frequency**	**Dosing timepoint**
Patient				

#### 2.11.2. Active tDCS

**Figure 2:**
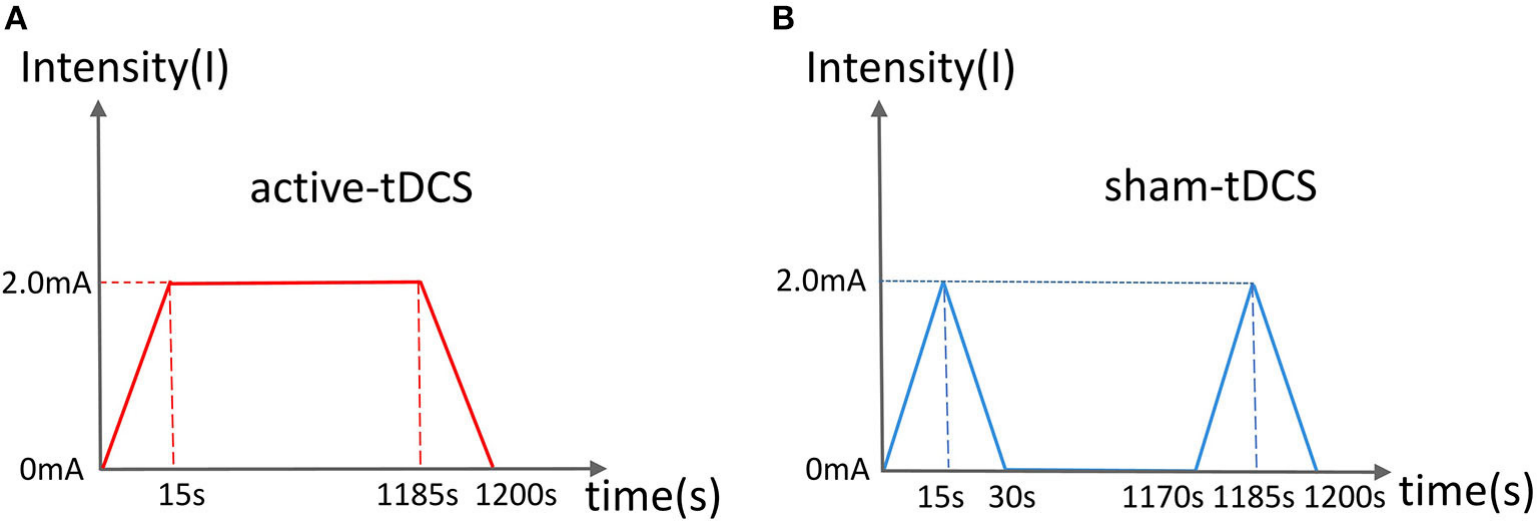
Current schematic diagram of active **(A)** and sham **(B)** tDCS. **(A)** The current rises and falls for 15 s, and lasts for 20 min. **(B)** The current rises and falls for 15 s and lasts for 1 min.

Participants in the intervention group will receive active tDCS treatment for PPD. The anode of the electrode will be placed on the left dorsolateral prefrontal cortex (dlPFC), and the cathode on the upper edge of the right track. Based on a previous professional study, the current strength will be set at 2.0 mA (Vigod et al., 2019). The circular electrode area size will be set at 35 cm^2^ (5 × 7 cm). The duration of the treatment will be set at 20 min per session, once a day for 6 days each week (Figure 2A). The participants will be treated with tDCS only, and will not be simultaneously treated using other methodsI. The treatment will be performed in a neuromodulation room at a designated time by a designated therapist.

#### 2.11.3. Sham tDCS

in the control group will receive sham tDCS treatment. The anode of the electrode will be placed on the left dlPFC and the cathode on the upper edge of the right track. During the first minute, the current will be input every 30 s, after which there will be no current output throughout the remaining 19 min of the sham treatment (Figure 2B). The remaining parameters will be similar to those of the active group.

### 2.12. Study endpoints

The endpoints of this study are as follows:

(1) If a severe adverse reaction occurs during the intervention process, it will be terminated promptly to protect the participant.

(2) The intervention will be terminated if a serious complication or worsening situation occurs during the intervention.

(3) The intervention will be terminated if the participant requests to withdraw, does not accept the treatment, or does not cooperate.

(4) If the therapist repeatedly explains that the treatment approach is not working, the study will be suspended.

(5) The intervention will be terminated if the subjects have suicidal intentions, mental disorders, treatment for sudden mania, or acute maternity complications throughout and following treatment (as determined by the study team).

Participants will have autonomy over their involvement in the study and can discontinue at any stage. The researchers will record in detail the reasons why and when participants drop out of the study. Participants who discontinue the treatment early will be included in an intention-to-treat analysis, which can be used to obtain follow-up information if the participants are willing.

## 3. Outcome assessment

The main outcome measures for the study are feasibility, accessibility, and compliance with the trial protocol. Feasibility describes how well the trial protocol can be implemented. We record data on practicability related to eligibility (for example, the number of psychiatric visits and percentage of eligible postpartum psychiatric hospital patients), recruitment (such as the number of eligible patients, screening methods, reasons for non-involvement), and timing (including the time participants started treatment). We also ask patients with PPD whether they can distinguish which treatment (active tDCS or sham tDCS) they have received at the end of the procedure during the study period to assess the feasibility of our blinding methods. Accessibility refers to patients' satisfaction with and opinions on the intervention, focusing on maternal, neonatal, and infant outcomes, and on whether they experience any effects or adverse outcomes. Acceptability measures include participants' vital signs monitored during each session, the effects or adverse outcomes monitored at the end of each week of the study period, and questionnaires to assess maternal outcomes. Aiming to improve the precision of self-reported assessments of these health indices, we provide patients with PPD with a duplicate of the relevant questionnaire. Finally, we will take some measures to assess compliance, including the number of sessions attended during the overall and expected active study period, the number of patients who completed the 15 active study periods, the reasons for dropping out, and the proportion of follow-up data collection.

### 3.1. Primary outcomes

The primary outcome will be the severity of depression symptoms evaluated by the MADRS (Montgomery and Asberg, 1979), an item with 10 parts to evaluate depressive symptoms. It is one of the most extensively used tools for assessing the degree of PPD in clinical practice (Fernandes et al., 2019). Every item of the MADRS is scored on a seven-point scale (from 0 to 6). The higher the score, the more severe the depression, with a total score of 0–60. A total score of ≥35 indicates extreme depression, 30–34 indicates major depression, 22–29 indicates moderate depression, and 12–21 indicates mild depression (Hudgens et al., 2021).

### 3.2. Secondary outcomes

The Perceived Stress Scale (PSS) (Cohen et al., 1983) is a commonly used self-report tool for evaluating the extent to which situations in an individual's life are rated as stressful. The items involved in this scale are devised to assess the extent to which respondents found their lives unpredictable, uncontrollable, and overloaded in the past month. The PSS includes 14 items on a five-point scale ranging from 0 (never) to 4 (very often), with a total score of 0–56. Higher scores indicate increased levels of perceived stress. The European Spanish version of the PSS will be used in the study (Remor, 2006).

The Positive and Negative Affect Scale (PANAS), which provides an assessment of these affects, will be used in this study (Watson et al., 1988). Participants will be asked to rate the degree of each emotional state they experienced on a Likert-type scale ranging from 1 (very mild or not at all) to 5 (exceedingly or very severe), resulting in a total score ranging from 10 to 50.

### 3.3. Follow up

After the three-week treatment, an eight-week follow-up will be performed. The participants will be contacted by medical staff by phone or in person. Additionally, participants will be contacted every 2 weeks to document their medication and recovery. During the final week of the follow-up process (week 8 of post-intervention), the participants can be referred for a clinical evaluation to evaluate their prognosis and their status of disability.

### 3.4. Statistical methods

The results of the test will be analyzed using the Statistical Package for the Social Sciences, version 21.0. In the descriptive analysis of the samples, continuous variables with normally distributed data will be expressed as means and standard deviations whereas continuous variables with non-normally distributed data will be expressed as medians and interquartile ranges. Statistical comparisons of normally distributed data packages between *t*-test groups will also be performed. Ordinary-level variables with non-normal distributions will be compared statistically using the Mann–Whitney *U* test. Metrics with discrete distributions will be expressed as percentages and analyzed using χ^2^ or Fisher's exact test, as applicable. The general linear or logistic models will be used if needed to adjust the obfuscation effect.

To compare the baseline characteristics between the intervention group and the control group, the *t*-test or Mann–Whitney *U*-test will be used. If statistically significant, the inequalities will be considered for confounding factors in the final efficacy analysis. To compare one or two outcomes between the two groups, continuous data will be analyzed using the *t*-test or the non-parametric test, and the data will be parsed using the Pearson χ^2^ test or Fisher's precision test. To control for possible confounding variables, linear models or linear regression models will be used for dependent continuous and dependent categorical variables. Repeating measure data will be used in the analysis of variance. The primary and secondary outcomes analyses will depend on intention-to-treat (ITT) and per-protocol (PP) bases. The results from ITT analyses will be compared with those from PP analyses to determine whether they are consistent. Missing data will be used as the final bearer for observation. The side effects will be displayed and parsed using a χ^2^ test or Fisher's precision test.

### 3.5. Safety monitoring

Safety and side effects will be evaluated daily throughout the active period of the study and at each follow-up and will be conventionally censored by the chief researchers. The study investigator team will provide the researchers with collective expertise in brain stimulation. Researchers will evaluate and audit safety during this pilot study. Mental health problems that require urgent medical attention will be conveyed to the researchers. Such mental health problems include self-destructive intention, homicidal intent, concern about child harm, evidence of psychotic symptoms, or any other related mental health issues based on the researchers' judgment. Urgent medical requests from participants will be treated appropriately to ensure their wellbeing. In such cases, the researchers will terminate the session (if this occurs during treatment), keep the participant in the office, and contact the psychiatrist for further directions. The psychosomatic medicine specialist will immediately evaluate the patient as required, with input from a specialist in maternal medicine. If no imperative medical or significant mental health conditions are found to be supported by this clinical evaluation, the participant will be directed to their psychosomatic care supplier, and, if necessary, the participant will be instructed to receive emergency services. When a participant's adverse event is recorded over the phone, emergency services will be contacted. Adverse events will be recorded and immediately reported (by telephone or fax within twenty-four hours) to the research ethics committees of both institutions to consider serious action, for example, the suspension of the intervention, the withdrawal of the participant, or the termination of the study.

## 4. Discussion

tDCS is a novel, noninvasive therapeutic approach for treating PPD and has the potential to introduce a safe, effective, low-cost, and accessible method of treatment. The study protocol presented in this paper is a pilot RCT that aims to guide the development of a larger multi-center study on determining the efficacy and safety of tDCS as a treatment option for PPD. The main site of tDCS is located at the left dlPFC, as the prefrontal cortex (PFC) has extensive and close relationships with the center and the remaining regions of the brain. Additionally, the PFC plays a key role in organizing, guiding, regulating, and processing information received within the brain, thereby making the overall function of the central nervous system harmonious (Abdelnaim et al., 2020). Regarding the treatment of PPD, tDCS stimulates the dlPFC through anodic stimulation to enhance the excitability of this area and other cortical connections to regulate anxiety and depression. Another possible mechanism of action is related to neurotransmitters. A related study (Luscher et al., 2011) found that tDCS could significantly increase the levels of GABA, DA, and 5-HT, thereby decreasing the levels of depression. GABA is a major inhibitory neurotransmitter in the central nervous system. Alterations in GABA signaling are associated with severe depressive disorders (Kalueff and Nutt, 2007). Furthermore, the antidepressant effects of tDCS may occur through direct or indirect regulation of DRN 5-HT neurons. A study in 2020 established that an acute cathode of tDCS induces the activation of prolactin cortical neurons and the inhibition of DRN 5-HT neurons, the amplitude of which are higher than that of acute tDCS of the same intensity (Cambiaghi et al., 2020). All the mechanisms of tDCS make it an ideal therapeutic approach for treating PPD.

Currently, there is a growing body of evidence on the effectiveness of tDCS as a treatment for PPD. In 2016, Sreeraj et al. (2016) used bipolar stimulation to treat a patient who was 6 weeks pregnant and had long-term depression. The anode was placed in the dlPFC of the brain. The cathode was placed in the F4 area, with 2 mA of current introduced for a period of 30 min over 10 days. Aside from the transient burning sensation at the treatment site during the initial stage, there were no other reported adverse reactions. The patient's Hamilton Depression Scale (HAMD) score will be measured during the one-month follow-up after treatment. The scores of the HAMD and those of the Hamilton anxiety scale dropped to six and five, respectively, indicating clinical remission, which is likely to be the first report of the successful use of tDCS among pregnant women experiencing depression. Vigod et al. (2019) conducted a three-week RCT study on the effectiveness of tDCS in the treatment of PPD. The treatment site was located at the dlPFC. Four weeks after the postpartum period, 75 percent of the patients with PPD symptoms were free of symptoms. Zhang et al. (2021) analyzed 27 RCTs, including 1,204 patients with depression, through meta-analysis, establishing that a stimulating current of 2 mA combined with a 30-min tDCS treatment course could enhance the curative effect. The extensive trials of tDCS in treating depression will play a significant role in formulating a protocol for evaluating the effect of this approach on PPD.

The subjective factors of patients can significantly influence the scales used in this study. The outcomes of the experiment are therefore susceptible to various factors. However, the scales used in this trial demonstrate excellent internal consistency, dependability, structural validity, and criterion validity and will be sensitive to alterations. In several conditions, these scales will be recommended for assessing fatigue levels. Therefore, we can enhance the precision of the experiments. However, the highly unstable electrode montage in the tDCS study may make the results inaccurate. The outcomes of a previous meta-analysis on the duration and length of tDCS sessions offer us an opportunity to further optimize this intervention (Labree et al., 2022). Relevant studies (Narita et al., 2017) also demonstrate the efficacy of the designed controlled trial for evaluating the effect of tDCS on the treatment of PPD.

Functional neuroimaging (FNI) techniques, such as functional magnetic resonance imaging, electroencephalography, and magnetoencephalography, are powerful tools for studying the human brain and laying methodological foundations for cognitive neuroscience (Li et al., 2019). Examining structural and functional changes in the brain through FNI can assist in making accurate clinical diagnoses among individuals experiencing PDD, potentially enhancing treatment approaches for this population and improving prognosis (Schnakenberg et al., 2021). However, compared to the relative wealth of data available for major depressive disorders, FNI-based studies focusing on PPD are limited in number and design (Fiorelli et al., 2015). A limitation that challenged the study was that consideration was not given to using FNI techniques among participants to determine the efficacy of tDCS in the treatment of PPD. Combining neuroimaging techniques and clinical data in subsequent studies to provide further insight regarding the treatment of PPD should be considered.

High-resolution modeling can be used to calculate the directional development of the spatial distribution of electric fields in the brain to understand how various stimulation parameters affect the current in neural tissues (Boayue et al., 2018). An et al. (2022) used personalized high-resolution virtual brain models to study individual network effects in deep brain stimulation among patients suffering from treatment-resistant depression, adding to the evidence for the use of high-resolution models to study depression. Additionally, Oshri et al. (2019) used high-resolution segmentation to reveal latent volumetric structures among amygdala nuclei and assessed the effects of adverse childhood experiences on amygdala morphometry as well as current psychiatric symptoms. This consolidated the results of previous studies linking early stress, right amygdala volume, and psychopathology. Therefore, high-resolution modeling is a crucial and powerful tool for the study of PPD treatment. In our future studies, we shall consider using high-resolution modeling on the magnitude of the induced electric field, and explore additional alternative regularization schemes to evaluate the effects of tDCS on the treatment of PPD.

Furthermore, because the experiments associated with this study will involve a small sample, in our future studies on tDCS, we shall strive to provide a novel medical foundation for treating patients with PPD using tDCS. As a result of the high prevalence and impact on patients' lives, tDCS plays a significant role in seeking additional effective treatment approaches for PPD. Additional tDCS trials or the treatment of PPD will provide unique insights into this subject. This trial is a meaningful exploration of enhancing the treatment for PPD. The number of PPD patients continues to increase, highlighting the importance of establishing effective and time-saving treatment techniques for this population. In the future, RCTs should be conducted on the premise of medical ethics and safety. Additionally, it is important that the screening of the target population follow clear and appropriate guidelines to avoid deviation of the experimental results. Increasing sample sizes along with the provision of exact experimental data can improve the likelihood of providing individuals with the best treatment methods available.

## Trial status

This trial was registered by the Chinese Clinical Trial Registry, ChiCTR2000029031. This study was registered on January 11, 2020.

## Ethics statement

The research study has been approved by the Ethics Committee of the First Affiliated Hospital of Nanchang University: Clinical Medicine Ethics Review [2015]043.

## Author contributions

WS, GY, and QZ designed the study. GL, XK, and MS wrote the manuscript. XD and ZZ collected the clinical data. KZ performed statistical analyses. All authors contributed to the editorial changes in the manuscript, read, and approved the final manuscript.
